# The flow of genetic information – the role of thyroid hormones in the regulation of gene expression

**DOI:** 10.1186/1756-6614-6-S2-A42

**Published:** 2013-04-05

**Authors:** Alicja Nauman

**Affiliations:** 1 Department of Biochemistry and Molecular Biology, The Centre of Postgraduate Medical Education, Warsaw, Poland

## 

Human genome encodes about 25 000 protein coding genes, among which some are constitutively expressed in all cells of the organism while the others are activated in response to specific extra- and intracellular signaling. Gene expression is regulated on multiple levels in a spatiotemporal manner. These levels include: genome, which contains all cellular DNA sequences (nuclear and extranuclear -mitochondrial), transcriptome, which contains all sequences of RNA synthesized in the cell (including long: pre-mRNA, mRNA, ncRNA, and short: snRNA, miRNA, and piRNA), proteome, which contains all proteins synthesized in the cell, metabolome, which includes all metabolic pathways in the organism, localizome, which includes all possible subcellular localizations of macromolecules in the organism, phenome, which is defined as a set of all phenotypes of the organism, which enables establishing of the real effects of particular genes and external environment on the phenotypic variability in humans.

Thyroid hormones, 3,5,3'5'-tetraiodothyronine (thyroxine) and 3,5,3'-triiodothyronine (T3) participate in the regulation of key cellular processes, including proliferation, differentiation, apoptosis and metabolism. Together with other factors they initiate variable cellular processes, starting from activation of the flow and amplification of genetic information (via DNA-RNA-protein), and ending with elicitation of specific phenotypic traits of the cell. The action of thyroid hormone, is mediated mainly by interactions with nuclear receptors (THR), which belong to the superfamily of ligand dependent nuclear transcription factors. THR bind to specific sequences of response to thyroid hormone (TRE) in promoters of target genes and regulate their expression. Apart from classic, genomic mechanism of action, THRs can also act via non-genomic mechanism in which regulation of target genes is mediated by interactions with other receptors – located in plasma membrane or cytoplasm. THR are encoded by two genes, THRA (ERBA) and THRB. These genes, located in chromosome regions 17q21 (THRA) and 3p21-3p25 (THRB), encode for three functional receptors: TRα1, TRβ1, and TRβ2, and the receptor TRα2 which does not bind T3 and acts as an antagonist of the other receptors. TRα1 and TRβ1 are expressed in all tissue types and their expression and ratios at which they are expressed depend on tissue type and developmental stage. In most tissues and organs only one of the receptors is responsible for T3 mediated regulation of gene expression. For instance, TRα1 is the main receptor acting in bones, TRβ1 acts in liver and kidney, while TRβ2 acts in pituitary and hypothalamus where it regulates HPT axis. In the genomic mechanism THR regulate expression of target genes via binding to the sequences of response to thyroid hormone (TRE) located in their promoters. Typical TRE consists of two repeats of AGGTC(C/A)A sequence. The presence of so called positive TRE results in induction of gene expression after binding of TR and T3, while binding of THR without T3 results in inhibition of gene expression. Thus, deficiency of T3 in the cell will result in inhibition of expression of a given gene, for instance GH or a gene coding for iodothyronine deiodinase type 1. The genes with negative TRE are regulated in an opposite manner: binding of T3 to the receptor results in inhibition of gene expression. The negative regulation of gene expression via T3 enables, among others, the mechanism of feedback regulation of expression of THR and TSH via T3. In consequence, all disturbances of thyroid hormone signaling pathways can directly result in deregulation of homeostasis of the organism.

